# Evaluating Fatty Acid Amide Hydrolase as a Suitable Target for Sleep Promotion in a Transgenic TauP301S Mouse Model of Neurodegeneration

**DOI:** 10.3390/ph17030319

**Published:** 2024-02-29

**Authors:** Shenée C. Martin, Kathryn K. Joyce, Kathryn M. Harper, Samuel J. Harp, Todd J. Cohen, Sheryl S. Moy, Graham H. Diering

**Affiliations:** 1 Department of Cell Biology and Physiology and the Neuroscience Center, University of North Carolina at Chapel Hill, Chapel Hill, NC 27599, USA; 2 Department of Psychiatry, University of North Carolina at Chapel Hill, Chapel Hill, NC 27599, USA; 3 Department of Neurology and the Neuroscience Center, University of North Carolina at Chapel Hill, Chapel Hill, NC 27599, USA; 4 Carolina Institute for Developmental Disabilities, Carrboro, NC 27510, USA

**Keywords:** sleep, Alzheimer’s disease, tau, endocannabinoids, anandamide, FAAH

## Abstract

Sleep disruption is an expected component of aging and neurodegenerative conditions, including Alzheimer’s disease (AD). Sleep disruption has been demonstrated as a driver of AD pathology and cognitive decline. Therefore, treatments designed to maintain sleep may be effective in slowing or halting AD progression. However, commonly used sleep aid medications are associated with an increased risk of AD, highlighting the need for sleep aids with novel mechanisms of action. The endocannabinoid system holds promise as a potentially effective and novel sleep-enhancing target. By using pharmacology and genetic knockout strategies, we evaluated fatty acid amide hydrolase (FAAH) as a therapeutic target to improve sleep and halt disease progression in a transgenic Tau P301S (PS19) model of Tauopathy and AD. We have recently shown that PS19 mice exhibit sleep disruption in the form of dark phase hyperarousal as an early symptom that precedes robust Tau pathology and cognitive decline. Acute FAAH inhibition with PF3845 resulted in immediate improvements in sleep behaviors in male and female PS19 mice, supporting FAAH as a potentially suitable sleep-promoting target. Moreover, sustained drug dosing for 5–10 days resulted in maintained improvements in sleep. To evaluate the effect of chronic FAAH inhibition as a possible therapeutic strategy, we generated FAAH−/− PS19 mice models. Counter to our expectations, FAAH knockout did not protect PS19 mice from progressive sleep loss, neuroinflammation, or cognitive decline. Our results provide support for FAAH as a novel target for sleep-promoting therapies but further indicate that the complete loss of FAAH activity may be detrimental.

## 1. Introduction

Highly conserved sleep behavior is tightly linked to higher cognitive functions, such as learning and memory. Acute and chronic disruptions in sleep can lead to and exacerbate neurodegenerative disorders, such as Alzheimer’s disease (AD) [1]. Our previous work in transgenic Tau P301S (called PS19) mice highlighted the link between sleep disruption and AD, as these mice exhibited progressive sleep loss and hyperarousal, tau pathology, neuroinflammation, and cognitive decline with age [2]. Several studies support sleep as an early biomarker [1,2,3,4,5,6,7] of AD progression, though it remains unclear how targeting sleep in AD can lead to positive outcomes in disease onset and progression. 

Endocannabinoids (eCBs) are endogenous bioactive lipids that signal through cannabinoid receptors, namely CB1 and CB2 [8,9]. CB1 receptors populate regions of the brain affected by Alzheimer’s disease, including the frontal cortex and hippocampal region [10], highlighting its potential relevance to disease. Anandamide (AEA), the first discovered endocannabinoid, strongly binds CB1 receptor, while 2-arachidonoylglycerol (2-AG) is more promiscuous and activates both CB1 and CB2 receptors [11]. Endocannabinoids are synthesized on demand to regulate neurotransmitter release and facilitate neuroprotection via anti-inflammatory and anti-excitatory signaling [11]. AEA and 2-AG are inactivated by separate enzymes, namely fatty acid amide hydrolase (FAAH) and monoacylglycerol lipase (MAGL), respectively, suggesting distinct regulation and physiological functions for each eCB metabolite. Additionally, since AEA and 2-AG are degraded by different enzymes, they can be independently targeted using selective pharmacological tools. The endocannabinoid system has functions in both cognitive and immune health, which support its relevance as a suitable treatment target. Additionally, endocannabinoids play a role or are influenced by the sleep/wake cycle [12,13]. We and others have shown that the activation of CB1 signaling, through a direct agonist or by enhancing the levels of AEA or 2-AG, can promote sleep [14]. The recent characterization of synapse proteome remodeling during the wake/sleep cycle shows that synaptic levels of FAAH are reduced during the sleep phase in mice [15]. Accordingly, using targeted lipidomics, we showed that the AEA metabolite and related FAAH substrates are upregulated during sleep [14]. Additionally, we have shown that AEA mediates homeostatic scaling-down in in vitro models, a synaptic plasticity type implicated in the restorative actions of sleep [15,16,17]. Taken together, we rationalized that FAAH/AEA are of particular relevance to the beneficial actions of sleep function. While several studies have shown that the manipulation of AEA, FAAH, or CB1 can increase sleep, few studies have investigated whether increasing sleep in AD will alleviate disease symptoms. 

To investigate the suitability of FAAH/AEA as a novel sleep-promoting therapeutic target in PS19 Tauopathy/AD mice models, we selectively targeted AEA via the acute and sustained administration of the FAAH inhibitor compound PF3845 and measured sleep phenotypes. PF3845 is well characterized and highly selective and can maintain AEA levels over a longer period of time compared to other FAAH inhibitors [18]. We confirmed PF3845 selectivity by mass spectrometry in both sexes [14]. Acute treatment increased sleep duration and reversed dark phase hyperarousal. The sleep-promoting effects of PF3845 were sustained over 5–10 days of oral dosing. Finally, we generated FAAH knockout (KO)/PS19 mice to test whether the long-term loss of FAAH and increased levels of AEA would be beneficial and feasible. We report that FAAH KO was not able to protect PS19 mice from progressive sleep loss and hyperarousal, neuroinflammation, or cognitive decline. Thus, while FAAH holds some promise as a suitable sleep-promoting target, complete loss of FAAH activity may not be beneficial.

## 2. Results

### 2.1. Dysregulation of the Endocannabinoid System in P301S Tau Mice

**Figure 1:**
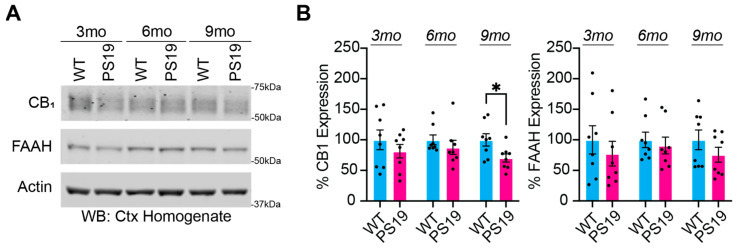
PS19 mice have decreased CB1 and FAAH expression. (**A**) Western blot analysis of cortical homogenate samples showing CB1 and FAAH in WT and PS19 mice at 3, 6, and 9 months. (**B**) Quantification of cortical CB1 and FAAH proteins in WT and PS19 mice at 3, 6, and 9 months. N = 8 per genotype. * *p* < 0.05. Unpaired two-tailed Student’s *t*-test. Error bars indicate ± SEM.

Previous studies report lower expressions of eCBs and the CB1 receptor in AD models [10]. A lower expression of CB1 may lead to reduced anti-inflammatory responses and accelerate AD pathogenesis. There is also evidence to suggest a decrease in FAAH activity in AD patients; however, it is unclear how this modulation by eCBs contributes to sleep disruption and pathology. PS19 Tauopathy/AD mice models are well characterized to exhibit age-related progressions in Tau pathology, neuroinflammation, cognitive decline, neurodegeneration, and atrophy [2,6,19,20,21,22]. Disease progression in PS19 mice can be broadly described as pre-symptomatic at 3 months of age, early symptomatic at 6 months, and late-stage disease at 9–11 months. We have recently shown that PS19 mice of both sexes exhibit a progressive loss of sleep as an early symptom that precedes and accelerates cognitive decline [2]. We hypothesized that PS19 mice exhibit abnormal expressions of CB1 and FAAH that progress with disease pathology. To examine the expression levels of CB1 and FAAH, we collected and homogenized whole cortices from PS19 mice and wild-type (WT) littermates at 3, 6, and 9 months old (Figure 1). Compared to the age-matched littermate controls, CB1 expression was significantly decreased in the 9-month-old PS19 mice (Figure 1B), with a non-significant trend to decrease at earlier age points. The FAAH expression data showed trends of decreased expression in the PS19 mice, though they were not significant (Figure 1B). This finding supports the use of treatments to increase CB1 signaling.

### 2.2. Selective Acute Inhibition of FAAH Promotes Sleep in P301S Tau Mice

**Figure 2:**
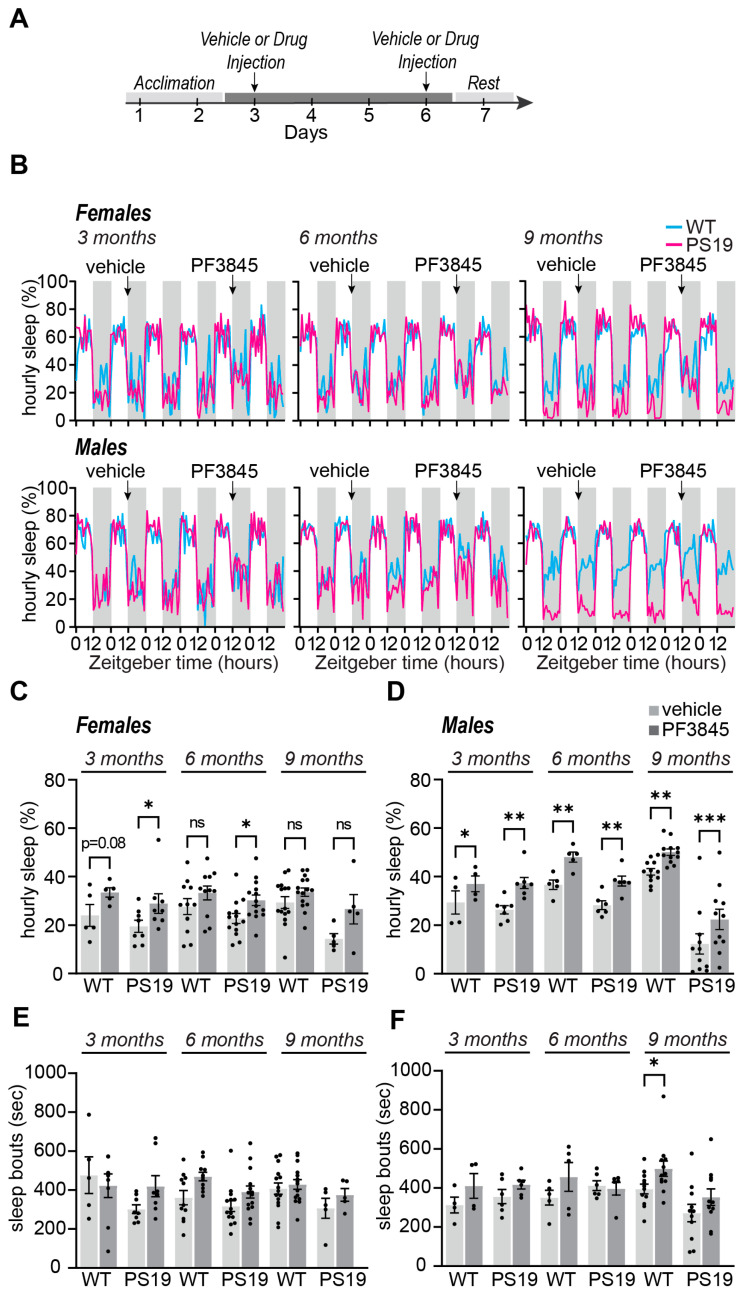
Selective increased AEA promotes dark phase sleep behavior in PS19 mice. (**A**) Experimental design; (**B**) 24 h traces of hourly sleep of female and male WT (blue line) PS19 (pink line) mice at 3 months (pre-pathology), 6 months (early phase), and 9 months (symptomatic phase). Grey bars in sleep traces indicate dark phase. (**C**,**D**) Quantification of average hourly dark phase sleep in females (**C**) and males (**D**). (**E,F**) Quantification of average dark phase sleep bout length in seconds in females (**E**) and males (**F**). N = 5–16/age/sex/genotype. * *p* < 0.05, ** *p* < 0.01, *** *p* < 0.001, ns: not significant. Paired two-tailed Student’s *t*-test. Error bars indicate ± SEM.

Previous studies have shown sleep modulation resulting from eCB manipulation [9,12]. Specifically, AEA has been highlighted for its role in sleep modulation [12]; therefore, we hypothesized that the pharmacological manipulation of AEA using a FAAH inhibitor would have sleep-promoting effects in PS19 mice, specifically acting to reverse the dark phase hyperarousal phenotype we have described [2]. Based on our previously published work [14], the selective inhibitor PF3845 (10 mg/kg) was used to block FAAH [18,23] (Figure 2). Sleep behavior was monitored using the PiezoSleep non-invasive piezoelectric home-cage monitoring system for the duration of the experiment (Figure 2B). This system has been validated with simultaneous electroencephalography (EEG) [24] and is highly suited for multi-day continuous recordings of sleep/wake behavior (12 h:12 h light dark cycle). We performed acute intraperitoneal (IP) injection at the onset of the dark (wake) phase (Zeitgeber time, ZT12) in 3-, 6-, and 9-month-old WT and PS19 male and female mice (Figure 2A). All mice received one vehicle and one PF3845 IP treatment separated by three days. We then measured the effects of the IP treatment during the immediate 12 h of the dark phase, directly comparing the results of the vehicle and PF3845 treatment for each animal. Consistent with our previous report, compared to the vehicle control, the acute PF3845 treatment exerted significant dark phase sleep-promoting effects in the WT males, but not in the females, at 3, 6, and 9 months (Figure 2B,D). The biological basis of this male-specific effect is not known. In PS19 females, the PF3845 treatment significantly increased the total dark phase sleep amount at 3 and 6 months, with a non-significant increasing trend at 9 months (Figure 2C). This suggests that the sleep-promoting effects of PF3845 can be seen in females when their sleep levels are reduced or fragmented compared to the WT. The PF3845 treatment significantly increased the dark phase sleep amount in the PS19 males at all ages (Figure 2D). The acute dark phase PF3845 treatment had a minimal effect on the subsequent light phase sleep behavior (Supplemental Figure S1A,B). Sleep bout lengths were largely unchanged in both sexes and genotypes (Figure 2E,F and Supplemental Figure S1C,D). These results suggest that inhibiting FAAH activity may be effective in increasing sleep time and reducing the dark phase hyperarousal phenotype seen in PS19 mice. Importantly, sleep-promoting effects could be seen at both the early and late stages of disease progression. These results also highlight the importance of sex-specific early intervention as an important variable in treatment development. 

### 2.3. Selective Sustained Inhibition of FAAH Promotes Sleep in P301S Tau Mice

**Figure 3:**
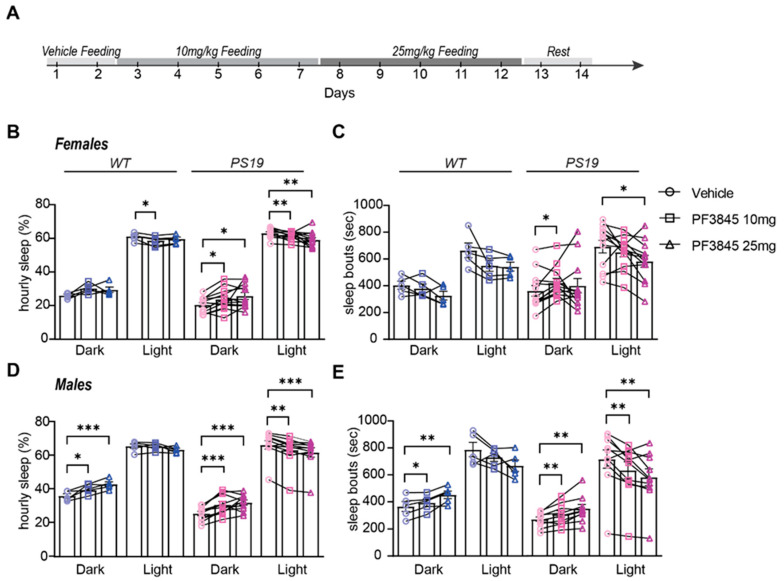
Sustained increase in AEA promotes dark phase sleep behavior in PS19 mice. (**A**) Experimental design. (**B**,**C**) Quantification of average hourly sleep (**B**) and sleep bout length (**C**) in female WT and PS19 mice. (**D**,**E**) Quantification of average hourly sleep (**D**) and sleep bout length (**E**) in male WT and PS19 mice. Data separated into 12 h of dark and light phases. N = 5–12/sex/genotype. * *p* < 0.05, ** *p* < 0.01, *** *p* < 0.001. Repeated measure two-way ANOVA with Šídák’s multiple comparisons test. Error bars indicate ± SEM.

While our previous results highlight the potential of PF3845 as a novel sleep-promoting agent, it is important to determine whether this effect can be sustained over longer treatment periods. Therefore, we designed an experiment to test the efficacy of a 10-day sustained administration of PF3845 in WT and PS19 male and female mice at 6 months. It was also important to test whether PF3845 exerts sleep-promoting effects when administered orally. The WT and PS19 mice were housed in our sleep recording facility for a 12-day recording of sleep/wake behavior. Following an acclimation period of ~24 h, the mice were fed a small quantity of peanut butter containing DMSO (dimethyl sulfoxide) (vehicle) at the dark phase onset on the first and second baseline (vehicle) days. For the next 10 days, the mice were fed at the dark onset with peanut butter containing PF3845 dissolved into DMSO; specifically, they were fed for 5 days at 10 mg/kg (low dose) and for another 5 days at 25 mg/kg (high dose) (Figure 3A). Peanut butter was found to be a highly palatable vehicle as it was readily consumed in less than 5 min by all of the examined mice without exception. Thus, this oral dosing regimen should be interpreted as a single bolus dosage. The sleep amount and bout lengths were quantified; the effects of the two PF3845 dosages were averaged across 5 days of treatment and compared to the averaged 2 days of vehicle treatment for each animal. The immediate effects in the dark phase and subsequent light phase were analyzed separately. Consistent with our previous results, the WT females did not show an increase in the dark phase sleep amount with either dose of PF3845. In contrast, the PS19 females showed an increased dark phase sleep amount after both PF3845 doses (Figure 3B), whereas only the low dose resulted in an increase in the dark phase sleep bout lengths (Figure 3C). Both the WT and PS19 males showed increased sleep amounts and sleep bout lengths in the dark phase with an apparent dose effect (Figure 3D,E). In both male and female PS19 mice, sustained PF3845 treatment resulted in secondary light-phase decreases in sleep amount and bout length, which is an effect that was not observed following a single acute dose (Supplementary Figure S1). Together, these findings suggest that sleep disruption in PS19 mice may be driven in part by impairments in eCB signaling and that targeting eCB signaling (increasing AEA) may be effective in increasing sleep time and quality and reducing hyperarousal. Additionally, these results highlight the potential suitability and sustainability of oral PF3845 dosing; however, more research is needed to understand the mechanisms of action and long-term consequences. 

### 2.4. Lack of Sleep-Promoting Effects of FAAH Knockout in P301S Tau Mice

**Figure 4:**
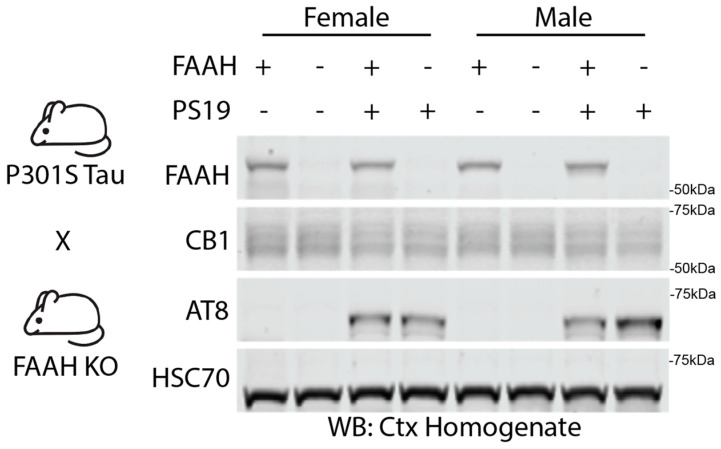
Validation of PS19/FAAH KO. Western blot analysis of cortical homogenate samples showing FAAH, CB1, and AT8 in FAAH+/−, FAAH−/−, FAAH+/−/PS19, and FAAH−/−/PS19 mice at 9 months.

The acute and sustained pharmacological manipulation of the eCB system may be a suitable approach for sleep promotion (Figure 2 and Figure 3). However, neurodegenerative conditions often progress over many years or even decades. Therefore, it is likely that effective treatments will need to be maintained chronically over long periods. In order to examine the suitability of the chronic loss of FAAH activity as a possible therapeutic approach, we examined whether the complete genetic knockout (KO) of FAAH would protect PS19 mice from progressive hyperarousal, neuroinflammation, and cognitive decline. Consistent with the effects of acute pharmacological FAAH inhibition, FAAH KO mice have been shown to have sustained increases in brain AEA and increased NREM sleep amount compared to controls [25]. We obtained FAAH−/− mice from Dr. Aaron Lichtman (Virginia Commonwealth University) and generated FAAH+/−, FAAH−/−, FAAH+/−/PS19, and FAAH−/−/PS19 cohorts for an experimental analysis. Figure 4 shows the Western blot expression profiles for FAAH+/−, FAAH−/−, FAAH+/−/PS19, and FAAH−/−/PS19 males and females at 9 months. The expression of FAAH was limited to FAAH+/− and FAAH+/−/PS19 animals, while AT8 expression, a marker of pathologically phosphorylated Tau, was only seen in FAAH+/−/PS19 and FAAH−/−/PS19 animals (Figure 4). 

**Figure 5:**
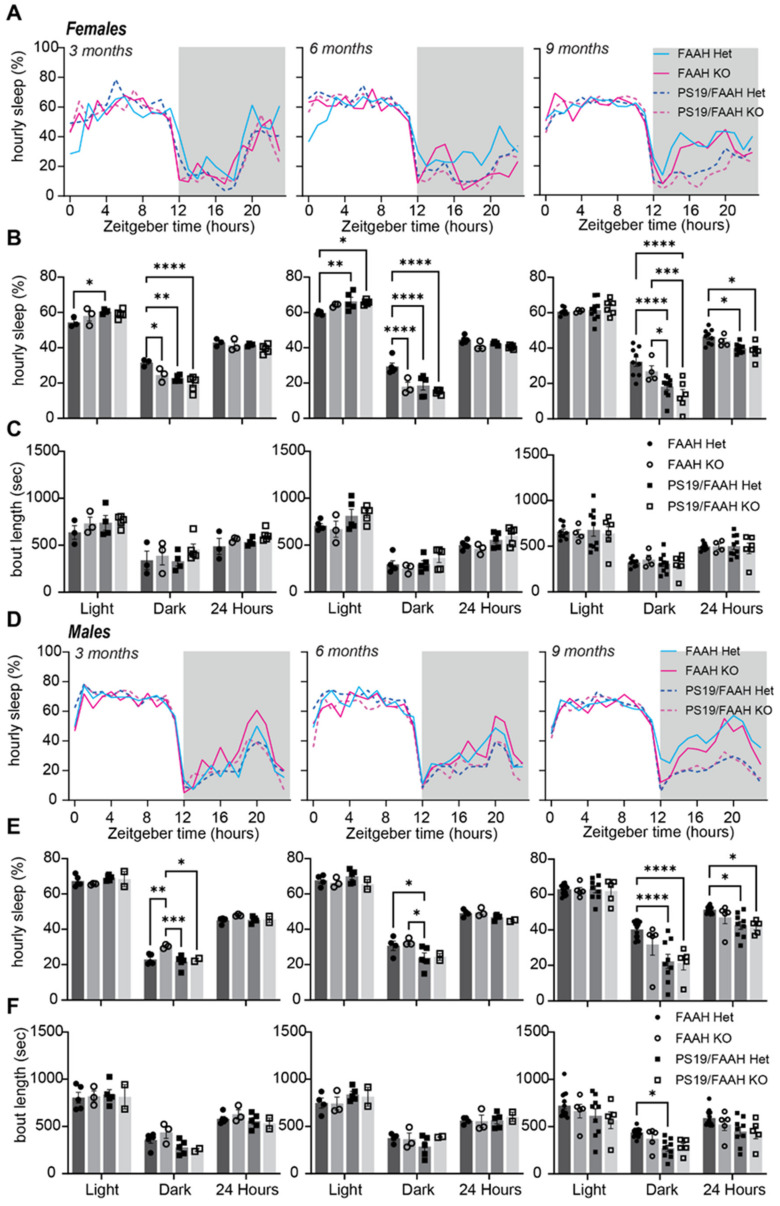
Loss of FAAH does not protect against sleep loss in PS19 mice; (**A**) 24 h trace of average hourly sleep of female FAAH+/− (blue line), FAAH−/− (pink line), FAAH+/−/PS19 (blue dashed line), and FAAH−/−/PS19 (pink dashed line) mice at 3, 6, and 9 months. Grey bars in sleep traces indicate dark phase. (**B**,**C**) Quantification of average hourly sleep (**B**) and sleep bout length in seconds (**C**). Data separated into 12hrs of dark and light phases; (**D**) 24 h trace of average hourly sleep of male FAAH+/− (blue line), FAAH−/− (pink line), FAAH+/−/PS19 (blue dashed line), and FAAH−/−/PS19 (pink dashed line) mice at 3, 6, and 9 months. (**E**,**F**) Quantification of average hourly sleep (**E**) and sleep bout length in seconds (**F**). Data separated into 12 h of dark and light phases. N = 5–17/age/sex/genotype. * *p* < 0.05, ** *p* < 0.01, *** *p* < 0.001, **** *p* < 0.0001. Two-way ANOVA with Šídák’s multiple comparisons test. Error bars indicate ± SEM.

Using our non-invasive sleep recording system, we measured the sleep amount and bout lengths in male and female FAAH+/− or FAAH−/− mice with or without the PS19 transgene at 3, 6, and 9 months of age (Figure 5). Approximately 6 days of uninterrupted sleep behavior were recorded and analyzed at each age. As we have observed previously, PS19 mice showed a progressively reduced dark phase sleep amount compared to the control littermates (FAAH+/− in this case), and by 9 months of age, female and male PS19 genotypes showed a significantly reduced daily 24 h sleep amount (Figure 5B,E). Contrary to our expected results based on prior works in the literature and the effects of pharmacological inhibition, FAAH KO did not protect PS19 mice from exhibiting age-related hyperarousal and sleep loss, which was particularly notable at 9 months of age (Figure 5B,E). These results indicate that the conventional genetic inhibition of FAAH may not be protective against hyperarousal in PS19 males and females nor promote sleep. FAAH inhibition may require a more targeted approach for conditional deletion after the transition to adulthood.

### 2.5. FAAH KO Does Not Protect against Inflammation in P301S Tau Mice

**Figure 6:**
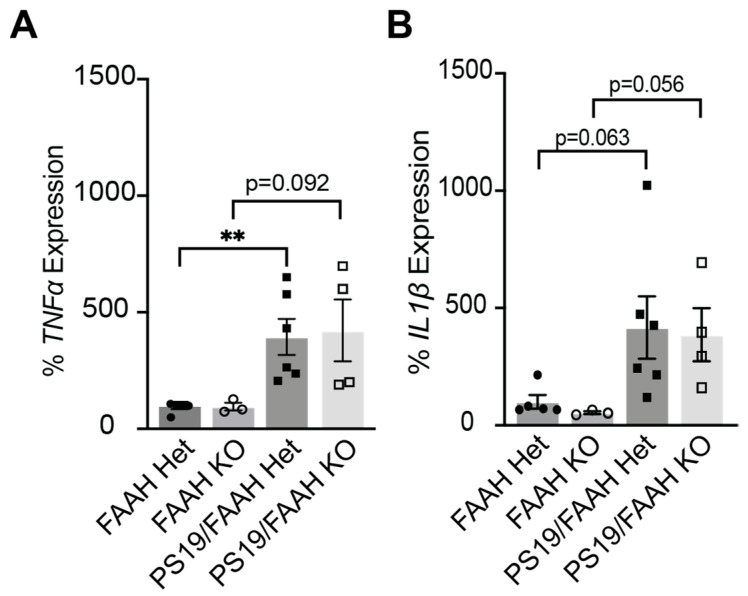
Pro-inflammatory cytokines are increased with loss of FAAH. (**A**) Quantification of *TNFα* mRNA levels in male and female FAAH+/−, FAAH−/−, FAAH+/−/PS19, and FAAH−/−/PS19 mice at 9 months. (**B**) Quantification of *IL-1β* mRNA levels in male and female FAAH+/−, FAAH−/−, FAAH+/−/PS19, and FAAH−/−/PS19 mice at 9 months. N = 3–6/genotype. ** *p*-value < 0.01; other p values are indicated. Unpaired Student’s *t*-test was performed. Bonferroni corrected. Error bars indicate mean ± SEM.

Neuroinflammation in the brain is another pathological hallmark of AD. Although neurons are especially vulnerable to the misfolding and aggregation of proteins in AD, microglia and astroglia are activated and trigger an innate immune response, releasing inflammatory factors that contribute to disease progression and severity [26]. Inflammatory cytokines IL-1β and TNFα have been shown to be increased in AD models [27] and around Aβ deposits [28] and increased in patients at risk for conversion from mild cognitive impairment to AD [29]. One major function of eCB signaling is the protection against inflammation. Thus, we next examined the relationship between FAAH KO and inflammation in FAAH+/−, FAAH−/−, FAAH+/−/PS19, and FAAH−/−/PS19 mice. We hypothesized that inflammatory cytokine expression would be reduced in PS19 mice with FAAH KO compared to mice that express FAAH. Whole cortical RNA was isolated from 9-month-old mice, and the *IL-1β* and *TNFα* mRNA levels were quantified by RT-qPCR. As expected, *TNFα* was significantly increased in FAAH+/−/PS19; *IL-1β* showed a similar increase that was not significant (Figure 6A,B). Counter to our expectations, the knockout of FAAH in the PS19 model did not show evidence of reduced inflammation levels (Figure 6A,B). These findings suggest that FAAH KO is not protective against inflammation in PS19 mice. 

### 2.6. FAAH KO Does Not Protect P301S Tau Mice against Cognitive Decline

**Figure 7:**
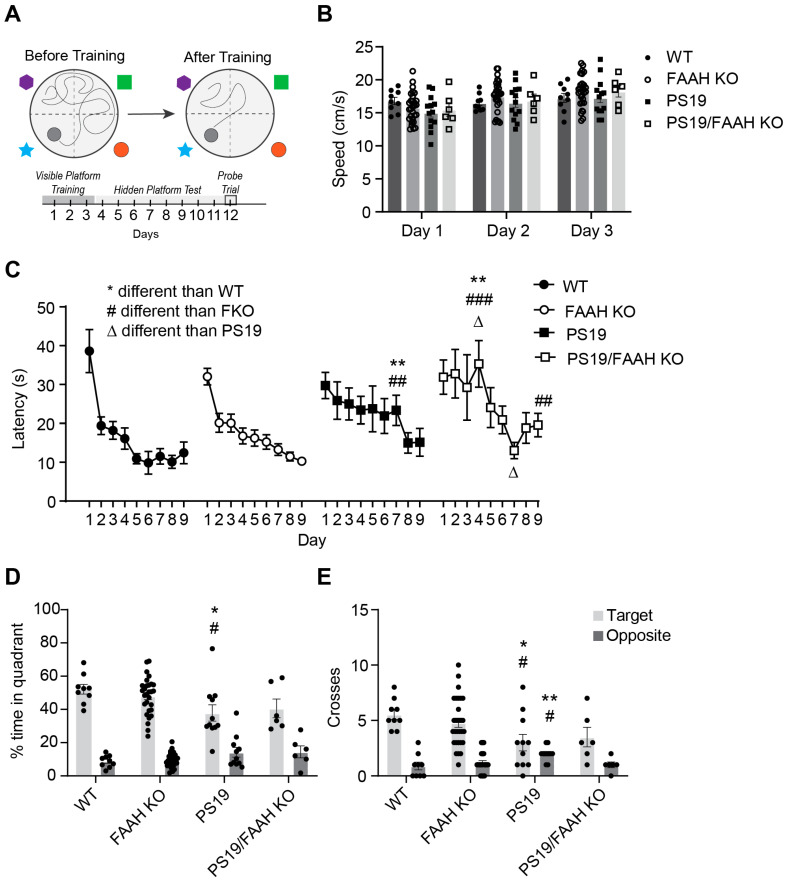
Loss of FAAH does not protect against cognitive decline in PS19 mice. (**A**) Experimental design. (**B**) Swim speed in WT, FAAH−/−, PS19, and FAAH−/−/PS19. (**C**) Acquisition of spatial learning. Escape latencies during acquisition in WT, FAAH−/−, PS19, and FAAH−/−/PS19 mice. (**D,E**) Spatial memory retention during 1 min probe trial, % time in target or opposite quadrant (**D**) or number of crosses of the target location (**E**) in WT, FAAH−/−, PS19, and FAAH−/−/PS19 mice. Target indicates the quadrant where the platform had been located versus the opposite quadrant. N = 6–29 per group (genotype, treatment). Data are shown as means (± SEM) of 4 trials per day in a Morris water maze. * *p* < 0.05, ** *p* < 0.01, comparison to WT. # *p* < 0.05, ## *p* < 0.01, ### *p* < 0.001, comparison to KO. Δ *p* < 0.05, comparison to PS19.

PS19 AD mice models have been shown to exhibit cognitive decline at the later stages of disease progression at ~9 months of age, most notably in hippocampal-dependent tasks of learning and memory [30,31]. Therefore, to test the hypothesis that FAAH KO would protect against cognitive decline in PS19 animals, we conducted the Morris water maze (MWM) test of spatial learning and contextual/acoustic cue fear conditioning to investigate hippocampal-dependent learning and memory. Behavior cohorts containing WT; FAAH−/−; PS19; and FAAH−/−/PS19 mice of both sexes were generated over several months and aged to about 8 months before the start of behavioral testing. In this experimental dataset, we were unable to generate sufficient numbers of experimental animals to conduct a satisfactory comparison between sexes; therefore, in this behavior dataset, we analyzed the results independent of sex. At the start of MWM testing, the mice were first placed in the water pool with a visible platform to assess if each individual was physically capable of completing the task and to measure the average swim speed as a simple readout of motor function (Figure 7A). All mice were able to locate the visible platform, and no individuals were excluded from further testing (not shown). Moreover, the average swim speed was found to be highly comparable between genotypes (Figure 7B). In the subsequent phase of the test, the escape platform was hidden below the surface of the water, and the mice were trained over 9 days to learn the location of the hidden platform. As expected, the WT mice showed a clear decrease in escape latency with training, which is indicative of spatial learning, and decreased escape latency was highly comparable in the FAAH−/− mice (Figure 7C). Both the PS19 and FAAH−/−/PS19 groups showed delayed acquisition, which is indicative of mild learning impairments in these genotypes. Following acquisition, the mice were given a one-minute probe trial with the platform removed from the pool to test for memory retention. As shown in Figure 7D, all groups had a preference for searching the target quadrant versus the opposite quadrant in the maze. However, the PS19 mice spent significantly less time in the target quadrant in comparison to the WT and FAAH−/− groups (Figure 7D). The PS19 mice also made fewer swim path crossings over the target location and more crossings over the incorrect opposite location than either the WT or FAAH−/− mice (Figure 7E). The FAAH−/−/PS19 mice did not show significant differences to the other genotypes, but overall, the performance of this group was highly comparable to the PS19 group, suggesting that the deletion of FAAH did not substantially improve cognitive performance. 

**Figure 8:**
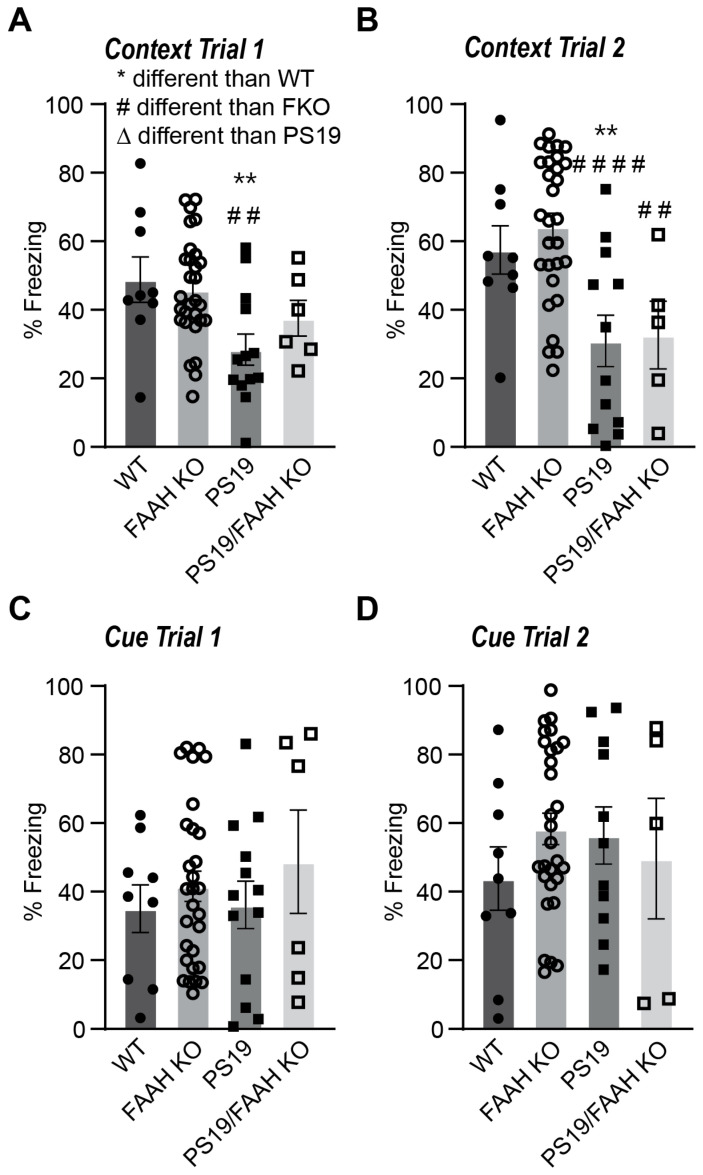
Loss of FAAH leads to differences in contextual learning, but not cue-dependent learning. (**A**,**B**) Percent freezing in the first 5 min context test (Test 1) and test 2 completed 2 weeks later in WT, FAAH−/−, PS19, and FAAH−/−/PS19 mice. (**C**,**D**) Percent freezing after an 80-decibel acoustic stimulus (3 min) was presented 2 min after mice were placed in the modified conditioned fear chambers (**C**). Cue test 2 was conducted 2 weeks following test 1 (**D**). N = 6–29 per group (genotype, treatment). ** *p* < 0.01, comparison to WT. ## *p* < 0.01, #### *p* < 0.0001, comparison to KO.

Finally, we examined memory performance in the contextual/cued-fear conditioning task. During training, the mice were placed into the fear conditioning apparatus, where they received three mild foot shocks paired with an acoustic stimulus. A total of 24 h later, the mice were returned to the fear conditioning context (without further stimulus), and freezing behavior was measured as a test of contextual memory retention, which is known to be hippocampus-dependent. A total of 48 h after training, the mice were placed in a novel context and presented with the acoustic stimulus, and freezing behavior was measured as a test of cued memory (hippocampal independent). Two weeks after training, contextual and cued memories were tested a second time as measures of long-term memory retention. Based on previous experiments in our PS19 mice, we expected to see deficits in contextual fear conditioning rescued in the FAAH−/−/PS19 mice. Genotype differences were observed during the first and second tests for contextual learning (Figure 8A,B). In both tests, the PS19 mice had significantly lower freezing than the WT or FAAH−/− mice. The FAAH−/−/PS19 mice showed a strong trend of a reduced freezing time in contextual trial 1, comparable to the PS19 mice, and showed a significant reduction in freezing during context trial 2, which is indicative of impaired long-lasting contextual memory (Figure 8A,B). All groups had comparable performance in the two tests for cue-dependent learning (Figure 8C,D). This experiment clearly indicates that PS19 mice exhibit age-related decline in hippocampal memory performance, and this decline was clearly not improved by the deletion of FAAH. 

## 3. Discussion

Although our knowledge of mechanisms underlying neurodegenerative diseases has grown, the lack of curative treatments for AD remains problematic. Sleep disturbances are reported at an alarming rate in AD patients and are now highlighted as a possible early biomarker in AD pathogenesis [6,21,32]. Several studies have described sleep loss as sufficient to induce neuroinflammation, AD pathology, and cognitive decline [33,34,35]. A compelling study found that chronic short sleep (CSS) induced synapse loss, tau pathology, and inflammation a year after CSS treatment in WT animals [33]. Current sleep aids do not perpetuate and enforce the restorative benefits of sleep. Interestingly, eCBs play a role or are influenced by the sleep/wake cycle [12,13]. Additionally, the eCB system functions in both cognitive and immune health, which further supports its relevance as a suitable treatment target. However, it is not clear how sleep promotion via targeting eCBs in AD translates to beneficial therapeutic intervention.

Previously, we reported a significant hyperarousal phenotype, defined by increased wakefulness in the dark phase, in PS19 males and females that worsens with age and pathological burden [2]. We also showed cognitive decline resulting from chronic sleep disruption [2]. Therefore, in the current study, we examined whether the pharmacological or genetic inhibition of FAAH would increase sleep and rescue cognitive decline in PS19 mice. We show that the acute inhibition of FAAH increased the total sleep time in the dark phase at 3, 6, and 9 months in PS19 mice. Based on these results, we then asked whether the sustained inhibition of FAAH at 6 months of age would lead to an increase in sleep. Sleep time in the dark phase was increased in PS19 males and females, which is in agreeance with our acute PF3845 result. We then hypothesized that the complete knockout of FAAH would rescue hyperarousal and cognitive impairment in PS19 mice. Indeed, FAAH KO mice have previously been reported to exhibit sustained increases in AEA and to engage in non-rapid eye movement (NREM) sleep more than WT mice [25]. Thus, we obtained FAAH−/− mice to genetically target the eCB system and crossed those mice with our PS19 mice. Surprisingly, the complete knockout of FAAH did not rescue hyperarousal, reduce neuroinflammation, or improve cognitive performance in the PS19 mice. Our results highlight FAAH as a potential therapeutic target in sleep promotion but caution against its complete knockout for long-term treatment. 

### 3.1. Endocannabinoids and Relevance for Sleep

Sleep behavior is highly complex and controlled by circadian (appropriate time of day) and homeostatic (sleep need) mechanisms. Previously published data have established that eCB metabolites may fluctuate with the sleep/wake cycle and maintain sleep, independent of the homeostatic sleep drive [9,12,13,36]. More specifically, our lipidomic data showed a significant increase in AEA and similar metabolites during sleep and after sleep deprivation in adolescent aged P56 mice [14]. Similar results were not seen in younger developing mice [14], indicating that AEA may be regulated by circadian mechanisms and functions to maintain and stabilize sleep during the transition to adulthood. We also investigated 2-AG, which was not increased and appeared to be expressed in a dissimilar pattern independent of the sleep/wake cycle [14]. These two metabolites are well characterized and independently degraded by separate enzymes. Our previous work shows that the selective inhibition of MAGL or FAAH resulted in increased 2-AG or AEA [14], respectively. Additionally, it has been shown that FAAH fluctuates with the sleep/wake cycle [15], further supporting its therapeutic potential in sleep promotion. 

Manipulations of the endocannabinoid system have proved informative on mechanisms of sleep behavior. Interestingly, the direct infusion of eCBs into the cerebral ventricles, hippocampus, or lateral hypothalamus has been shown to promote rapid eye movement (REM) sleep [37,38,39,40]. Additional studies highlight the role of eCBs in both NREM and REM sleep [9]. Utilizing pharmacological and genetic approaches, many studies have shown that the enhancement of eCB signaling promotes NREM stability, whereas the inhibition of eCB signaling causes NREM fragmentation [9,12,13,25,36,41,42]. Specifically, the inhibition of FAAH in the dark phase increased NREM stability; however, MAGL inhibition in the dark phase resulted in later fragmentation of sleep in the subsequent light phase [12]. Similarly, the activation of CB1 with a direct agonist immediately increased NREM stability but resulted in later disruptions in NREM amount and duration, which were suggested to result from a functional downregulation of CB1 following sustained increases in 2-AG [12,36]. The complete blockade of CB1 resulted in NREM fragmentation, highlighting the relevance for AEA investigation in sleep promotion. 

### 3.2. Sex-Specific Effects of Sleep Promotion

Sex- and genotype-specific differences were seen in the efficacy of PF3845 to improve sleep. Consistent with our previous publication, PF3845 treatment increased sleep amount in WT males at all ages, but not in WT females. We speculate that the sleep-promoting effects of PF3845 may only be apparent in female mice that exhibit reduced or disrupted sleep, whereas healthy levels of sleep in WT females may show a ceiling effect. The dichotomy between male and female responses to PF3845 may stem from inherent biological differences between males and females. There is no evidence to suggest a lower abundance of eCBs in males or females, as shown in our previous paper; however, we saw differing levels of several NAE species during sleep in adolescent males [14]. 

Male-specific differences could be due to receptor expression differences [43,44,45]. In AD specifically, the CB1 mRNA levels were shown to be increased in the 3xTgAD mouse model in the prefrontal cortex (PFC), dorsal hippocampus (DH), and basolateral amygdala complex (BLA) at 6 and 12 months of age [46]. However, the protein levels were decreased at 12 months in the BLA and DH [46]. It is also evident from our previous work that the sleep-promoting effects of FAAH inhibitors in males are blocked by the CB1 antagonist AM251 [14]. We hypothesize that the altered expression of CB1 in males is promoting the effects of PF3845 treatment and causing a larger response to treatment. Additional information is needed to further develop PF3845 as a therapeutic agent in both males and females.

### 3.3. Effects of Acute vs. Sustained FAAH Inactivation

Sleep disruption is becoming increasingly recognized as a possible biomarker for early disease progression in AD. Several studies report sleep disruption in different AD models and have established a bidirectional relationship between sleep disruption and AD-related pathology and cognitive decline [1,2,6,7,19,47,48]. Therefore, therapies aimed at protecting sleep during early stages of disease progression may be beneficial in delaying the onset of pathogenesis. Unfortunately, the long-term use of current front-line sleep aids is associated with an increased risk of developing AD [49,50]; thus, there is a need for sleep-based medicines with novel mechanisms of action. PF3845, an FAAH inhibitor, has been shown to be beneficial in reducing inflammation by suppressing pro-inflammatory gene expression [51]. PF3845 is also highly selective, and its effects are reversible [18]. Our previous findings agree with this study and show a selective increase in AEA abundance but not 2-AG after PF3845 treatment in both sexes [14]. 

The potential therapeutic benefits of PF3845 are exciting; therefore, we utilized this drug in PS19 mice. No negative consequences of PF3845 have been reported; however, it has been noted that PF3845 treatment in anxiety produces an anxiolytic-like response [52]. Additionally, PF3845 has a short half-life, which we confirmed with our acute PF3845 administration experiments. The effects of PF3845 were no longer present after 12 h post treatment. Acute PF3845 treatment increased dark phase sleep, countering the hyperarousal phenotype in PS19 males and females. Sustained daily dosing experiments also resulted in increased sleep and sleep bout lengths. Surprisingly, in our sustained dark phase PF3845 administration experiments, we noticed a subsequent significant decrease in light phase sleep that was prominent in PS19 males and females. Whether this decrease in light phase sleep is detrimental is unknown. It is possible that increased sleep during the dark phase consequent to PF3845 treatment naturally reduces the need for sleep in the subsequent light period. Another possibility is that the decrease in light phase sleep may be a secondary effect of the PF3845 treatment resulting from a delayed downregulation of CB1 following a period of elevated AEA signaling. Indeed, a secondary fragmentation of sleep has been reported following an initial increase and consolidation of sleep after treatment with an MAGL inhibitor (increased 2-AG) or direct a CB1 agonist [12]. Additionally, previously reported consecutive daily dosing of PF3845 (10 mg/kg) for 6 days was shown to provide sustained analgesia with no indication of tolerance or downregulation of cannabinoid receptors [53], unlike the effects of MAGL inhibition, which did not result in sustained analgesia. However, these results do not account for the effect on sleep behavior. We hypothesize that this may be compensation for the sleep gained in the dark cycle. Future studies are necessary to understand this phenotype and the mechanisms that may be driving light phase sleep reduction.

### 3.4. Evaluation of FAAH KO Model

FAAH hydrolyzes anandamide and also supports other lipid signaling to promote anti-inflammatory and analgesic responses [54]. Both in vitro and in vivo studies support FAAH’s role in the protection against anxiety [55,56,57,58], depression [57], and inflammation [59,60] without affecting cognition. FAAH inhibition also supports sleep by increasing anandamide availability, which is believed to play a role in the maintenance of sleep. Our previous results provided strong evidence for the potential of sleep promotion via FAAH KO. However, FAAH KO in PS19 mice did not rescue hyperarousal, neuroinflammation, or cognitive decline. One reason for this could be that the long-term loss of FAAH is detrimental during development. Studies have reported deficits in early pregnancy events [61] and inflammation-induced preterm birth [62], likely due to an imbalance of anandamide availability. FAAH KO mice exhibited a 15-fold increase in anandamide levels [25], and this sustained level of anandamide may perpetuate long-term consequences from development into adulthood. 

Endocannabinoids are produced on demand and function to promote homeostasis. The genetic inhibition of FAAH may lead to abundant anandamide availability and the desensitization or inactivation of CB1 receptors. One study compared the effect of chronic inhibition of MAGL and FAAH. MAGL blockade (pharmacological and genetic) led to the desensitization of CB1 and the loss of analgesic activity. Conversely, chronic FAAH inhibition via PF3845 did not lead to desensitization and maintained analgesic activity [53]. Although the pharmacological inhibition of FAAH is promising, we hypothesize that the complete loss of FAAH from birth diminishes the beneficial effects of anandamide by desensitizing the CB1 receptor, which then leads to sleep disruptions. Additionally, the loss of FAAH at a certain time of day may be beneficial, but a complete loss throughout the day is detrimental. FAAH has been shown to also fluctuate with the sleep/wake cycle [15], suggesting that FAAH inhibition may be time-sensitive. This is a limitation of the KO genetic model, as time-restricted inhibition would only be accomplished via pharmacology. 

Finally, endocannabinoids function in sleep homeostasis and maintenance. Higher amounts and quality of slow wave sleep were reported in the FAAH KO mice compared to their WT littermates [25]. We were not able to reproduce these results, as our FAAH KO mice did not show elevated sleep behaviors (Figure 5). FAAH KO also did not protect the PS19 mice from hyperarousal phenotypes nor increase sleep (Figure 5), which may have led to poor behavioral outcomes in the cognitive tests (Figure 6 and Figure 7). In our experience, we noted that some of our FAAH KO mice exhibited spontaneous and unexplained deaths. These data leave many questions surrounding the feasibility and efficacy of the complete knockout of FAAH from birth. Additional studies need to be conducted to investigate conditional knockout efficacy.

## 4. Materials and Methods

### 4.1. Mice

Animal procedures were all approved by the Institutional Animal Care and Use Committee of the University of North Carolina (UNC) and performed according to guidelines set by the U.S. National Institutes of Health. P301S (PS19) mice were obtained from a colony maintained at UNC and bred with C57BL/6J females purchased from Jackson Labs. C57BL/6J females were allowed to acclimate to the housing at UNC for at least 2 weeks before breeding. PS19 mice were bred with either WT or FAAH KO animals obtained from Dr. Aaron Lichtman (Virginia Commonwealth University) to generate experimental cohorts containing male and female WT, PS19, FAAH+/−, FAAH−/−, FAAH+/−PS19, and FAAH−/−/PS19 mice. Experimental animals were bred and housed in the animal facility until sleep behavioral analysis. Experiments were performed using 3-, 6-, and 9-month-old mice. Breeders (WT) for our colony were replaced every 3 months with mice supplied from Jackson Labs. FAAH+/− female breeders were maintained in house. 

### 4.2. Western Blot

Male and female 3-, 6-, and 9-month-old mice were sacrificed at Zeitgeber time (ZT) 4, after which whole mouse cortices were dissected in ice-cold phosphate-buffered saline. Brains were immediately frozen on dry ice and kept at −80 °C until further processing. Frozen mouse cortices were homogenized using 12 strokes from a glass homogenizer in ice-cold homogenization solution (320 mM sucrose, 10 mM 175 HEPES, pH 7.4, 1 mM 2,2′,2″,2″′-(Ethane-1,2-diyldinitrilo) tetraacetic acid [EDTA], 5 mM Na 176 pyrophosphate, 1 mM Na3 VO4, 200 nM okadaic acid [Roche]). For Western blot, proteins (5 ug) were separated via sodium dodecyl sulfate polyacrylamide electrophoresis (SDS-PAGE) on 10% acrylamide gel. After sample separation, proteins were transferred onto a nitrocellulose membrane (0.2 um Bio-Rad) and incubated with 3% Bovine Serum Albumin (BSA; Fischer bioreagents) blocking buffer (100 mm Tris pH 7.5, 165 mm NaCl; TBS) for 30 min at room temperature. Membranes were then incubated with primary antibodies (full list provided in Table 1) in a solution of 3% *w*/*v* BSA in TBST (100 mm Tris pH 7.5, 165 mm NaCl, 0.1% *v*/*v* Tween 20; TBST) at 4 °C overnight. Membranes were then probed with secondary antibodies with 1:15,000 IRDye 680RD-conjugated goat anti-mouse (LICOR Biosciences, Lincoln, NE, USA) and 1:15,000 IRDye 800RD-conjugated donkey anti-rabbit (LI-COR) in 3% *w*/*v* BSA in TBST for 1 h at room temperature. The immunoreactivity of all antibody signals was detected simultaneously with a Li-Cor Odyssey CLx IR imaging system (Li-Cor).

### 4.3. Sleep Monitoring and Behavior Analysis

PS19 mice were moved to our wake/sleep behavior satellite facility on a 12 h:12 h light/dark cycle (lights on from 7 a.m. to 7 p.m.). Mice were individually housed in 15.5 cm^2^ cages with bedding, food, and water and allowed to acclimate to their surroundings for at least two full dark cycles before the beginning of data collection and experimentation. No other animals were housed in the room during these experiments. As described in detail in our previous publications [2,14,63], sleep and wake behaviors were recorded using a noninvasive home-cage monitoring system, PiezoSleep 2.0 (Signal Solutions, Lexington, KY, USA). The system uses a Piezoelectric polymer sensor pad to transform mechanical signals, such as breath rate and movement, into electrical signals. Customized software (SleepStats version 4, Signal Solutions, Lexington, KY, USA) was used to interpret sleeping and waking patterns. The system can discern normal sleeping patterns of sleep based on typical sleeping behaviors of mice. 

### 4.4. Drugs and Treatments 

PF3845 (Cayman Chemicals, Ann Arbor, MI, USA) is a drug that inhibits the enzyme FAAH and is expected to drive a selective increase in the levels of AEA and related N-acylethanolamides (NAEs) [18]. PF3845 was dissolved in DMSO and then prepared into a vehicle solution (5% dimethyl sulfoxide [DMSO], 5% Kolliphor, 90% of a 1% NaCl solution) at the doses indicated. To assess the efficacy of FAAH inhibition on sleep behavior, PS19 and WT mice received an intraperitoneal (IP) injection immediately preceding the onset of the dark phase (ZT12). In our cross-over design, each mouse received an injection of a vehicle or drug followed by a second injection 72 h after vehicle or drug administration. These experiments were conducted in 3-, 6-, and 9-month-old PS19 and WT animals. Sleep behavior was examined for 24 h following each injection. The effects of the drug were determined by comparing the 24 h following drug injection to the 24 h following vehicle injection for each mouse.

For oral administration, PF3845 was dissolved and prepared into solution (5% dimethyl sulfoxide [DMSO], 5% Kolliphor, 90% of a 1% NaCl solution) at 10 mg/kg and 25 mg/kg [18]. Animals housed in the satellite facility were given 0.25 g of peanut butter mixed with varying doses of PF3845 (10 mg/kg, 25 mg/kg) mixed in by hand. Peanut butter was distributed randomly preceding the onset of the dark phase. PS19 and WT animals received peanut butter with DMSO (vehicle) for 2 days after the 1-day acclimation period, and then received the drug mixture for 10 consecutive days preceding the onset of the dark cycle. Sleep behavior was assessed throughout the experiment and at least 24 h after the last dose was given. 

### 4.5. Quantitative Real-Time PCR

The RNeasy Plus Mini kit (QIAGEN) was used to extract the total RNA according to manufacturer’s instructions. cDNA was obtained by reverse transcription of RNA using the High-Capacity cDNA Reverse Transcription Kit (Applied Biosystems, Waltham, MA, USA). QuantStudio 7 Flex (Applied Biosystems) was used with TaqMan Fast Advanced master mix to perform quantitative real-time PCR (RT-qPCR). Approximately 0.360 ug/uL was used for RNA analysis. Several TaqMan primer/probes (Invitrogen, Waltham, MA, USA) were used, including *GAPDH* (Cat. Mm99999915_g1), *TNF-α* (Cat.Mm00443258), and *IL1-β* (Cat. Mm00434228_m1). Expression data were normalized to FAAH+/− with *GAPDH* as the reference gene. All expression data follow the 2-ΔΔCt method with values calculated as fold gene expression. 

### 4.6. Behavioral Paradigm

Morris water maze. The water maze was used to assess spatial learning, swimming ability, and vision using published methods [64]. The water maze consisted of a large circular pool (diameter = 122 cm) partially filled with water (45 cm deep, 24–26 °C) and surrounded by numerous visual cues around the room. The procedure involved two phases: a visible platform test and acquisition in the hidden platform task.

Visible platform test. Each mouse was given 4 trials per day, across 3 days, to swim to an escape platform cued by a patterned cylinder extending above the surface of the water. For each trial, the mouse was placed in the pool at 1 of 4 possible locations (randomly ordered) and then given 60 s to find the visible platform. If the mouse found the platform, the trial ended, and the animal was allowed to remain on the platform for 10 s before the next trial began. If the platform was not found, the mouse was placed on the platform for 10 s, and then given the next trial. Measures of latency were taken to find the platform and swimming speed via an automated tracking system (Noldus Ethovision, Leesburg, VA, USA). 

Spatial learning in a hidden platform task. Following the visible platform task, mice were tested for their ability to find a submerged, hidden escape platform (diameter = 12 cm). Each mouse was given 4 trials per day, with 1 min per trial, to swim to the hidden platform, and testing was continued across 9 days. On the final day, mice were given a 1 min probe trial in the pool with the platform removed. Selective quadrant search was evaluated by measuring the percentage of time spent in the target quadrant (where the platform had been located) versus the opposite quadrant, and the number of swim path crosses over the target location where the platform had been placed during training versus the corresponding area in the opposite quadrant. 

Fear conditioning. Mice were evaluated for learning and memory in a conditioned fear test (Near-Infrared image tracking system, MED Associates, Burlington, VT, USA), using published methods [65]. The procedure had the following phases: training on day 1, a test for context-dependent learning on day 2, and a test for cue-dependent learning on day 3. Two weeks following the first tests, mice were given second tests for retention of contextual and cue learning.

Training. On day 1, each mouse was placed in the test chamber, contained in a sound-attenuating box, and allowed to explore for 2 min. The mice were then exposed to a 30 s tone (80 dB) that co-terminated with a 2 s scrambled foot shock (0.4 mA). Mice received 2 additional shock-tone pairings, with 80 s between each pairing. 

Context- and cue-dependent learning. On day 2, mice were placed back into the original conditioning chamber for a test of contextual learning. Levels of freezing (immobility) were determined across a 5 min session. On day 3, mice were evaluated for associative learning to the auditory cue in another 5 min session. The conditioning chambers were modified using a Plexiglas insert to change the wall and floor surface, and a novel odor (dilute vanilla flavoring) was added to the sound-attenuating box. Mice were placed in the modified chamber and allowed to explore. After 2 min, the acoustic stimulus (an 80 dB tone) was presented for a 3 min period. Levels of freezing before and during the stimulus were obtained by the image tracking system. Learning retention tests were conducted two weeks following the first tests. 

### 4.7. Statistical Analysis

All data from sleep behavior experiments were analyzed in Microsoft Excel (Windows Office 2021) or GraphPad Prism version 9.1.0 (GraphPad Software LLC, Boston, MA, USA). To satisfy our crossover design, each animal was compared to itself in the acute inhibition experiments to determine sleep phenotypes. Paired *t*-tests were used to compare vehicle to treatment in each animal. For sustained PF3845 experiments, a repeated measure two-way analysis of variance (ANOVA) with Šídák’s multiple comparisons test was used. Cognitive behavior experiments were conducted by experimenters blinded to mouse genotype. Statview (SAS, Cary, NC, USA) was used for data analysis. One-way or repeated measures ANOVA were used to determine effects of genotype. Post hoc comparisons were conducted using Fisher’s Protected Least Significant Difference (PLSD) tests only when a significant F value was found in the ANOVA. Within-genotype comparisons were conducted to determine quadrant selectivity in the water maze. For all comparisons, significance was set at *p* < 0.05.

## 5. Conclusions

Overall, our data highlight the efficacy of PF3845 as a sleep-promoting agent in a model with hyperarousal. We make a strong case for targeting FAAH as a useful therapeutic target but highlight the need for additional experiments to confirm its feasibility. We raise strong concerns about the complete knockout of FAAH from birth and speculate that loss of FAAH may lead to endocannabinoid signaling dysfunction, inflammation, and premature death. Our study provides evidence that FAAH may be a valuable target to promote sleep and treat disease; however, more research is required to identify the most appropriate regimen for targeting FAAH.

## Figures and Tables

**Table 1:** Western blot antibodies used in PS19 cortical homogenate analysis.

Antibody	Species	Source	Concentration
Anti-AT8	Mouse	Thermo Fisher UJ2859471	1:1000
Anti-CB1	Rabbit	Cell Signaling CST 93815	1:1000
Anti-FAAH	Mouse	Abcam AB54615	1:1000
Anti-Actin	Mouse	EMD Millipore MAB1501	1:10,000
Anti-Hsc70	Mouse	EMD Millipore MABE1120	1:1000

## Data Availability

The original contributions presented in the study are included in the article/supplementary material, further inquiries can be directed to the corresponding author.

## Supplementary Materials

The following supporting information can be downloaded at https://www.mdpi.com/article/10.3390/ph17030319/s1, Figure S1: Selective increased AEA does not promote light phase sleep behavior in PS19 mice subsequent to dark phase PF3845 dosing.
