# Supplementary material for: Evaluating Fatty Acid Amide Hydrolase as a Suitable Target for Sleep Promotion in a Transgenic TauP301S Mouse Model of Neurodegeneration
Source: Pharmaceuticals (Basel). 2024 Feb 29;17(3):319. doi: 10.3390/ph17030319 (PMC10975243; doi:10.3390/ph17030319)
Supplement: Supplementary file 1 [file pharmaceuticals-17-00319-s001.zip › pharmaceuticals-2861439-supplementary.pdf]

Supplementary Material

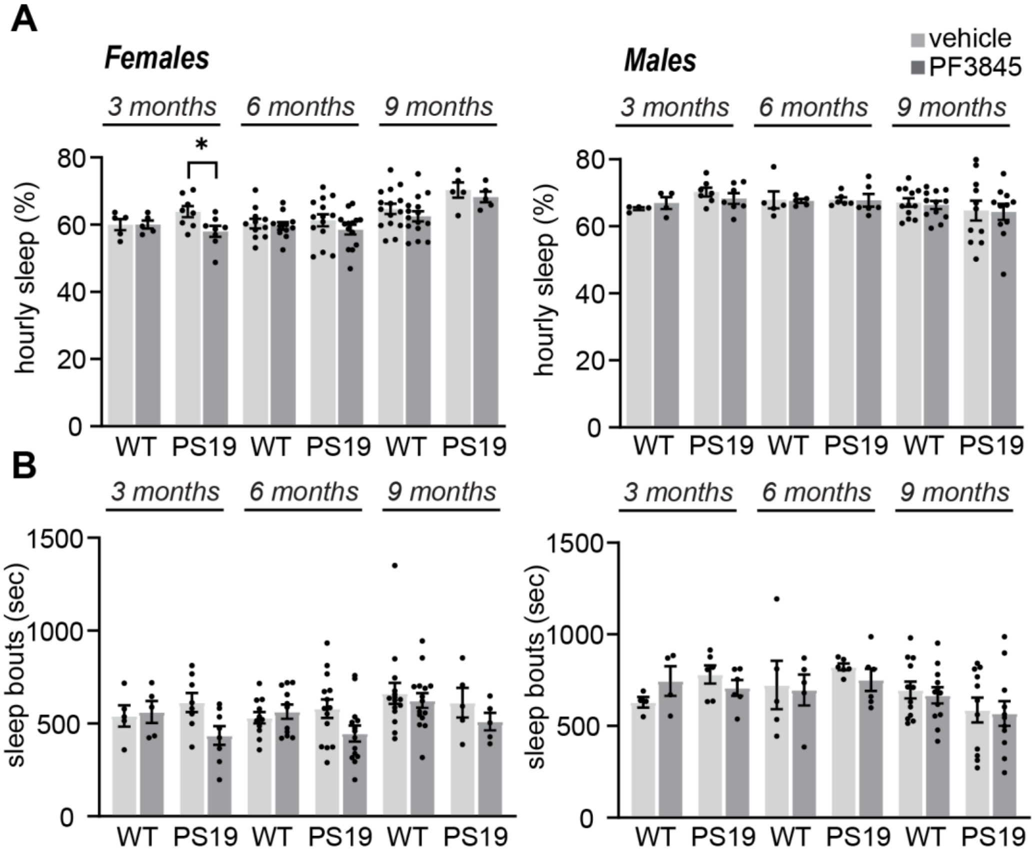

Supplemental Figure S1. Selective increased AEA does not promote light-phase sleep behavior in PS19 mice, subsequent to dark phase PF3845 dosing. (A) Quantification of average hourly light phase sleep in females and males. (B) Quantification of average light phase sleep bout length in seconds in females and males. N=5-16/age/sex/genotype. *$p<0.05$. Paired two-tailed student's t-test. Error bars indicate ± SEM.
